# Two Cases of Popliteal Aneurism Successfully Treated by Compression

**Published:** 1851-07

**Authors:** 


### ECLECTIC DEPARTMENT,

### AND SPIRIT OF THE MEDICAL PERIODICAL PRESS.

---

*Two Cases of Popliteal Aneurism successfully treated by Compression.* By James R. Wood, M. D., one of the Surgeons to Bellevue Hospital.

Case 1. John Morgan, aged thirty, born in Ireland, carpenter, admitted into Bellevue Hospital, December 29th, 1849. He is temperate in his habits, and has enjoyed good health. While at work, twelve months ago, the scaffolding on which he was standing gave way, and he fell to the ground, striking his left leg over the popliteal space on an upright piece of timber. Inflammation and swelling followed, and the part was very painful; under proper treatment, these symptoms disappeared in eight or ten days, leaving a small hard tumor, which has since remained, slowly increasing in size. This tumor is the size of a small orange, is nearly globular in shape, and has a very firm feel. Strong pulsations can be felt when the fingers are placed over it; these pulsations are synchronous with those of the heart, and cease when compression is made on the femoral artery above it; at the same time the tumor diminishes somewhat in size. A faint aneurismal thrill can be heard at times by applying the ear to the part; this ceases with the pulsations. Pulsation and the thrill return, and the tumor regains its size as soon as pressure is removed. Slight pain is sometimes felt in the affected part.

Commenced treatment January 23, 1850, by compression, using alternately Dupuytren's instrument and the thumb. Patient was able to bear the compression twenty-five minutes at the first application; second, thirty; third, thirty-five; the thumb being used in the intervals, which varied from ten minutes to half an hour. Very soon after the instrument was applied, the temperature of the limb fell two or three degrees, but in the course of three hours the natural heat was restored.

Jan. 24th. Compression has been constantly kept up since yesterday; patient is able to bear the instrument thirty minutes this morning without much pain; no pulsation felt in the tumor since half-past three o-clock this morning; treatment is borne well; no untoward symptom; pressure slightly moderated.

Jan. 25th. Treatment continued. Last night, at half-past 11 o,clock, patient complained of pain in his back, and was very restless; pulse sixty-three per minute, and moderately full; tongue clean: functions regularly performed. Ordered sulph. morph. one-sixth of a grain, after which he was in a great measure relieved, and slept three hours; bears treatment much better than yesterday; instrument borne an hour without pain; no pulsation.

Jan. 26th. Treatment still continued; pressure moderated so as to be borne three hours without inconvenience; leg slightly swollen; still of natural temperature; a branch of one of the internal articular arteries can be felt pulsating on the inner side of the knee. This branch is enlarged, and is the size of or larger than the radial artery. No pulsation has been felt in the anterior or posterior tibial arteries since treatment was commenced.

Jan. 27th. No change of consequence since yesterday; slight compression borne six hours without pain. Leg not so much swollen as yesterday; small arterial branches can be felt pulsating on both sides of the tumor.

Jan. 31st. No change since last date of consequence; the tumor has sensibly diminished in size; collateral circulation appears to be growing stronger. The arterial branch inside the knee has increased in size somewhat; the instrument is still kept on to make slight compression.

Feb. 1st. A graduated compress and roller bandage applied instead of the instrument. The roller was carried from the toes to the thigh. Patient allowed to walk a little.

Feb. 10th. Compress and roller still kept on; tumor very much diminished in size. The veins of the affected leg are larger than those of the other side. There seems to be some obstruction to the return of blood above the knee. Patient allowed to walk around the ward, and back and forward in the hall adjoining. He has no pain, but says the knee joint feels a little stiff, and that the foot feels numb sometimes. There is no pulsation yet felt in either of the tibial arteries.

Feb. 20th. All treatment discontinued except restriction in exercise; tumor nearly absorbed; a vestige merely remaining; veins still continue enlarged, but are not so much so as at last date. The sensation of numbness has almost entirely disappeared.

During the whole treatment the ordinary hospital diet has been allowed in moderate quantities. The appetite has been good, digestion and the other functions have been performed with perfect regularity.

I am indebted to my house-surgeon, Dr. Loving, and Dr. Loines, housesurgeon of the second surgical department, for their assistance in the treatment of this case.

Case 2. A—— B——, aged 33, N. Y., carman, intemperate, has had syphilis, for which he was treated with mercurials. He first noticed a small tumor behind the left knee, one month since, accompanied with slight pain, which he attributed to a violent jump. This did not trouble him much, and was almost forgotten, until at the end of the week, when another leap, and a slip upon a round stone, were followed by great pain, and steady increase of the swelling, which compelled him to apply for medical relief; the case was readily recognized by his physician, Dr. Leveridge, but on account of the influence of his previous habits upon the nervous system, when he became confined to the house, treatment for the cure of the aneurism was delayed until this day, June 28, 1850. The tumor is at present larger than a goose-egg, the pain is great, shooting down the leg at each pulsation, and constantly enhanced by the excessive edema of the limb below the knee. General health, average; pulse, eighty-eight; respiration, twenty-four.

The plan of cure is, as in the case of Morgan, to interrupt entirely and constantly, the primary current of blood to the aneurism, by the use of Dupuytren's compressor to the middle of thigh, alternating with pressure by the thumb to the artery, at the rami pubis. With this view, pressure by the instrument was first applied at 9 o'clock forty minutes, A. M., and continued thirty minutes, when, on account of the pain caused by it, the circulation was confined by the thumb at the rami pubis, and the pressure by the instrument taken off twenty minutes, after which it was re-applied, and the assistants so directed to make the alternation for the day; also, to aid the return of blood by friction with the hand, when pressure by the instrument should be taken off, and the patient rendered more comfortable by keeping the leg flexed.

In a few minutes after the circulation was thus cut off, the leg and foot became cold, but the heat soon returned to the upper part of the leg, and the superficial veins soon became enlarged, but gradually diminished in size, 9 P. M., same day. Pressure has been steadily kept up as directed, more than eleven hours, at about the same alternations; about five hours since, the patient suddenly complained of excruciating pain in the knee and leg for a few minutes; and in the course of an hour from that time, pulsation could be felt in one of the internal articular arteries. The tumor is now hard and incompressible, and upon removing all pressure from the femoral artery no pulsation can be perceived in it; notwithstanding this, pressure directed to be continued, with somewhat less severity, in the same manner during the night, the leg to be enveloped in cotton, and an anodyne administered.

June 29th. 10 A. M. Pressure has been steadily continued; patient showed some symptoms of mania a potu. There is no pulsation in the tumor, although it can be felt in the artery to within an inch of the aneurism; the leg is still cold; pressure continued. June 30th, 9 A. M. Lighter pressure has been continued in the same way, and the patient now bears the instrument two hours at a time; the natural warmth has returned to the whole limb; anodynes are still administered at night; pressure to be continued. July 1st. All going on well; edema of the limb is subsiding, making the tumor more definable. July 2d. Same note as yesterday; pressure is needed to be maintained so slightly, that the instrument is continued three or four hours in one place, and then a little up or down the thigh; the application of the thumb has not been needed since last night. July 4th. All going on well; up to this time an assistant has remained constantly with the patient; but now it is considered unnecessary for the attendance of an assistant; no pulsation can be felt in the femoral artery below the tendinous arcade of the triceps. July 6th. All going on well; the instrument is still kept on to confine the patient to the bed.

July 20th. Absorption is going on in the tumor, the edema is subsiding; patient is recovering the use of the limb, and he is allowed to sit up and move about on crutches. The whole limb is bandaged, and graduated compresses are applied along the course of the femoral artery. Pulsation can be felt in numerous small vessels about the knee, but it may be worthy of noting here that at no time since the patient has been under treatment could pulsation be detected in either of the tibial arteries.—*N. Y. Jour. of Med.*

